# Constitutive Phenotypic Modification of Lipid A in Clinical Acinetobacter baumannii Isolates

**DOI:** 10.1128/spectrum.01295-22

**Published:** 2022-07-21

**Authors:** Su-Hyun Kim, Sohyeon Yun, Woojun Park

**Affiliations:** a Laboratory of Molecular Environmental Microbiology, Department of Environmental Science and Ecological Engineering, Korea University, Seoul, Republic of Korea; University of Manitoba

**Keywords:** carbapenem-resistance, polymyxin, lipopolysaccharide, phosphoethanolamine, *pmrC*, Gram-negative bacteria

## Abstract

The degree of polymyxin B (PMB) resistance was measured in 40 clinical Acinetobacter baumannii isolates obtained from health care facilities. All of the tested isolates possessed a multidrug-resistant (MDR) phenotype against four classes of antibiotics (meropenem, doxycycline, gentamicin, and erythromycin), except for PMB. The *bla*_OXA-23_ gene was detected throughout the genetic analysis and experimental assay, indicating that all of the MDR strains were carbapenem-resistant A. baumannii strains. Multilocus sequence typing-based genotyping revealed that nine selected strains belonged to the international clone II lineage. When matrix-assisted laser desorption ionization–time of flight mass spectrometry was performed, intrinsic lipid A modification by phosphoethanolamine (PEtN) incorporation was noticeable only in the PMB-resistant (PMB^R^) strains. However, the presence of hexa- and penta-acylated lipid A due to the loss of the laurate (C_12_) acyl chain was noted in all PMB-susceptible strains but not in the PMB^R^ strains. The reduction of negative surface charges in the PMB^R^ strains was assessed by zeta potential analysis. Fluorescence imaging using dansyl-PMB revealed that, in the PMB^R^ strains, PMB was less likely to bind to the cell surface.

**IMPORTANCE** The widespread presence of MDR pathogens, including A. baumannii, is causing serious hospital-acquired infections worldwide. Extensive surveillance of MDR clinical A. baumannii isolates has been conducted, but the underlying mechanisms for their development of MDR phenotypes are often neglected. Either lipid A modification or loss of lipopolysaccharide in Gram-negative bacteria leads to PMB^R^ phenotypes. The prevalence of intrinsic lipid A modification in PMB^R^ clinical strains was attributed to high levels of basal expression of *pmrC* and *eptA-1*. Our findings suggest that new therapeutic strategies are warranted to combat MDR pathogens due to the emergence of many PMB^R^ clinical strains.

## INTRODUCTION

Acinetobacter baumannii is a nosocomial opportunistic pathogen that is regarded as a threat to human health because of the increase in the prevalence of its multidrug-resistant (MDR) phenotypes (1). Globally, medical infections caused by pathogenic A. baumannii strains accounted for 2 to 4% of clinical cases in 2016; however, MDR strains were found to be responsible for up to 45% of all A. baumannii infections, probably because of poor antibiotic stewardship (2). Carbapenem, a front-line antibiotic, has often been used for the treatment of MDR strain-linked infections in many clinical situations when third-generation broad-spectrum cephalosporins, such as cefixime, cefotaxime, and ceftazidime, are not appropriate (3). However, the high incidence of carbapenem-resistant A. baumannii (CRAB), primarily by the spread of *bla*_OXA-23_, has been attributed to a strong need for last-line therapeutic options for the treatment of CRAB infections (4). Polymyxins, which are cationic antimicrobial peptides (CAMPs), are less efficacious *in vivo* and induce nephrotoxicity in humans; however, they are considered the last treatment option for infections caused by many carbapenem-resistant MDR Gram-negative bacteria, including CRAB strains (5). Polymyxin B (PMB) and polymyxin E (colistin) antibiotics act on lipid A, a constituent of lipopolysaccharide (LPS). They replace the divalent cations (e.g., Mg^2+^) of lipid A cross bridges and lead to lytic cell death as a result of weakened membrane integrity and uncontrolled leakage of cellular components (6).

The outer membrane (OM) bilayer of Gram-negative bacteria has unique lipid asymmetry and harbors selective pores in inner-leaflet phospholipids and outer-leaflet LPS, serving as a robust barrier against toxic compounds, including antibiotics (1). Although canonical lipid A of LPS has a bisphosphorylated disaccharide of d-glucosamine (GlcN) as its major backbone, many bacterial species have distinct acyl chain numbers and lengths because of the differences in their lipid A biosynthetic pathways (typically with four to seven acyl chains, as follows: tetra-acyl, *Helicobacter* and *Yersinia*; penta-acyl, *Rhodobacter*; hexa-acyl, Escherichia, Klebsiella, and Salmonella; hepta-acyl, Acinetobacter) (7). The number and chemical structure of acyl chains of lipid A can also change under different environmental conditions, which can affect membrane fluidity, virulence, and the activation of the mammalian innate immune system (e.g., Toll-like receptor 4 [TLR4]/myeloid differentiation protein 2 [MD2]) (8). Because of symmetrical and amphipathic OM structures, the OM has less fluidity and poorer permeability than the inner membrane (IM); this prevents hydrophobic antibiotics, such as chloramphenicol and aminoglycosides, from entering cells. However, the biophysical properties of the OM and LPS linked to membrane fluidity remain unclear (9). The second stage of binding of cationic PMB to lipid A after electrostatic contacts involves hydrophobic interaction between the fatty acid tail of PMB and acyl chains of lipid A in the OM, which facilitates the self-promoted uptake of polymyxins across the OM and subsequent cell death through further PMB-mediated pore formation in the IM (6). Thus, the reduction of acyl chain lengths and numbers in lipid A increases membrane fluidity but weakens the interaction with PMB by reducing lipid A hydrophobicity, thereby making the bacteria less susceptible to PMB (10). However, a recent study demonstrated that lipid A is present in the periplasmic leaflets of the IM and surface of the OM and that PMB-mediated IM damage can lead to cell lysis and death during the active division of Escherichia coli (11). PhoPQ-induced PagL (Salmonella enterica and Pseudomonas aeruginosa) and PmrAB-induced LpxR (S. enterica and Yersinia enterocolitica) on the OM have been experimentally proven to be deacetylases working on existing lipid A; however, no corresponding protein has been characterized in the genomes of A. baumannii so far (1, 12, 13). Interestingly, the upregulation of a newly found LpxS of A. baumannii was found to transfer a short octanoate (C_8_:0) acyl chain to replace laurate (C_12_) at the C2′ position of lipid A under cold-stress conditions, thereby increasing membrane fluidity (14).

Deacylation and GlcN modification (with phosphoethanolamine [PEtN], galactosamine [GalN], or 4-amino-4-deoxy-l-arabinose [l-Ara4N]) in lipid A are controlled by many two-component systems (TCSs), such as PmrAB and PhoPQ, which can confer PMB resistance to cells (15). Environmental stimuli that activate TCSs provide fine-tuning gene regulation for bacterial adaptation under harsh conditions; however, uncontrolled TCSs as a result of the accumulation of mutations during adaptation in humans have often been reported in many clinical pathogenic bacteria (16–18). Constitutive activation of TCSs by mutation and recombination enables pathogens to survive under hostile host environments. Point mutations of a histidine kinase, BfmS (A42E/G347D, L181, or T242R [all cases in the sensor domain]), constitutively activate its downstream transcriptional regulator, BfmR, resulting in enhanced biofilm formation in clinical P. aeruginosa strains (19). In addition, mutations in the GraRS system of clinical Staphylococcus aureus can induce vancomycin resistance by activating the *dltABCD* operon and *mprF*, making it difficult to treat infections caused by the bacterium (20).

Frequent mutations in PmrB in the PmrAB TCS have been reported to account for a high PMB-resistant (PMB^R^) phenotype, possibly through the modification of lipid A, in several Gram-negative pathogens, such as E. coli, Klebsiella pneumoniae, P. aeruginosa, S. enterica, and A. baumannii (15). However, no lipid A analysis was conducted in the aforementioned cases. The transcriptional regulator PmrA activated by PmrB binds to the chromosomal promoter regions of the *pmrCAB* operon, the *arnBCADTEF* operon, and *naxD* in an unnamed operon, inducing the expression of *pmrC* (encoding a PEtN transferase), *arnT* (encoding an l-Ara4N transferase), and *naxD* (encoding an *N*-acetylgalactosamine deacetylase), respectively. However, the *arnBCADTEF* operon is not present in A. baumannii (21, 22). PEtN transferase encoded by either plasmid-driven *mcr* or insertion sequence (IS)-mediated *eptA* (a *pmrC* homolog) may be responsible for the spread of PMB resistance (23, 24). Interestingly, LPS deficiency caused by the loss of *lpx* has been reported to result in PMB resistance in several clinical A. baumannii strains (25, 26). In the present study, matrix-assisted laser desorption ionization–time of flight (MALDI–TOF) mass spectrometry (MS) was performed to analyze the compositions of lipid A in both PMB^R^ and PMB-susceptible (PMB^S^) MDR strains. Fluorescence images using dansyl chloride-labeled PMB (dansyl-PMB) and FM 4-64 were also obtained to visualize the binding of PMB to each cell. Amino acid variations in PmrC, PmrA, and PmrB were assessed via genomic and PCR analyses to elucidate the mechanisms underlying polymyxin resistance. Collectively, higher basal expression levels of *pmrC* and the consequent PmrC-mediated modification of lipid A with PEtN were monitored only in PMB^R^ clinical MDR A. baumannii strains.

## RESULTS

### PMB resistance in clinical A. baumannii isolates.

Antibiotic susceptibility tests of all of the tested strains were performed by assessing the MICs of five classes of antibiotics, including PMB (Table 1; also see Table S2 in the supplemental material). Of the 41 strains (40 clinical isolates and 1 laboratory wild-type [Lab-WT] strain), 82.5% (33/40 strains) appeared to be MDR strains, showing resistance to more than three antibiotics (Table 1; also see Table S2). The selected nine high MDR clinical strains showed much higher resistance to all of the tested antibiotics except for PMB. Hence, they were selected for further analysis (Table 1). For all nine strains, the MIC of each antibiotic was very high (e.g., doxycycline, 16 to 32 μg/mL; gentamicin, >512 μg/mL; erythromycin, 256 to 512 μg/mL), far exceeding the reference levels (Table 1). These MICs were much higher than many other reported MICs for clinical A. baumannii isolates (doxycycline, 0.25 to 64 μg/mL; gentamicin, 0.25 to 512 μg/mL; erythromycin, 0.5 to 4 μg/mL), indicating that our tested clinical isolates could have intrinsic antibiotic resistance and might have acquired multiple antibiotic resistance genes (ARGs) through mobile genetic elements (MGEs) (27–29). In the case of the A. baumannii NCCP 16007 genome, 32 functionally diverse ARGs [e.g., *mph*(E) [macrolide 2′-phosphotransferase], *armA* [aminoglycoside methyltransferase], *tet*(B) [tetracycline efflux pump], and *bla*_OXA-23_ [β-lactamase]] linked to MGEs were characterized in our previous report (30). Similar MICs for doxycycline (1 to 64 μg/mL) and meropenem (0.05 to 16 μg/mL) resistance have also been noted in other clinical pathogenic bacteria (K. pneumoniae, E. coli, and P. aeruginosa) (15, 31). Eighteen of the 40 clinical isolates exhibited high resistance to meropenem (>16 μg/mL), which belongs to the carbapenem family. Thus, a large proportion (45% [18/40 strains]) of clinical isolates could be considered CRAB (Table 1; also see Table S2). Notably, significant differences in the measured PMB MICs were observed among all 9 strains. The strains could be categorized into two groups based on their level of PMB resistance, i.e., PMB^R^ strains (128 to 256 μg/mL) and PMB^S^ strains (1 to 2.5 μg/mL) (Table 1). While all of the strains displayed high resistance to four different antibiotics, only 4 strains (NCCP 16007, NCCP 15996, NCCP 15995, and F-1629) demonstrated high resistance to PMB (128 to 256 μg/mL) (Table 1). Resistance to polymyxin, an antibiotic used as the last resort for treating CRAB infections, was noted in less than 10% of CRAB isolates in the Republic of Korea in the past decade; in the present study, however, a higher rate (22% [4/18 strains]) of PMB resistance was noted among CRAB isolates (Table 1; also see Table S2) (32). Genetic and biochemical differences were further analyzed using the two groups of MDR strains (PMB^R^ and PMB^S^).

**TABLE 1:** Antibiotic susceptibility tests of clinical A. baumannii isolates used in this study

Strain	Origin^*a*^	Year of isolation	Region of isolation	MIC (μg/mL) of:^*b*^				
				Polymyxin B^*c*^	Meropenem	Doxycycline	Gentamicin	Erythromycin
Reference strain								
ATCC 17978 (Lab-WT)	Laboratory			2	1	1	1	16
PMB^R^ strains								
NCCP 16007	Urine	2011	Seoul	256	16	32	>512	512
NCCP 15996	Urine	2013	Gyeonggi-do	256	64	32	>512	>512
NCCP 15995	NA	2013	Seoul	128	>64	32	>512	512
F-1629	NA	NA	Seoul	128	64	32	>512	>512
PMB^S^ strains								
NCCP 15989	Sputum	2013	Jeollabuk-do	2.5	64	32	>512	>512
NCCP 16006	NA	2011	Seoul	2	64	32	>512	512
NCCP 16011	NA	2011	Seoul	2	32	32	>512	512
NCCP 15992	NA	2013	Seoul	1	32	32	>512	>512
NCCP 15994	Pus	2013	Gyeongsangbuk-do	1	64	16	>512	256

a NA, not available.

b The MICs were measured using the 2-fold dilution method.

c The MIC of PMB was measured in a previous study (30).

### Genetic profiling of clinical A. baumannii isolates.

Unique allelic profiles of clinical A. baumannii isolates were analyzed according to the Oxford scheme using seven housekeeping genes, namely, *gltA*, *gyrB*, *gdhB*, *recA*, *cpn60*, *gpi*, and *rpoD* (33). The nucleotide sequence for multilocus sequence typing (MLST) was corrected using the draft genome sequence obtained using the NovaSeq 6000 platform (Illumina, USA). All of the tested A. baumannii strains, including the Lab-WT strain, were grouped into six different sequence types (STs) (ST112, ST208, ST357, ST358, ST369, and ST451) according to the PubMLST database for A. baumannii (https://pubmlst.org/organisms/acinetobacter-baumannii) (Table 2; also see Fig. S1). The dominant ST (ST357) contained 5 strains (NCCP 16007, NCCP 15995, F-1629, NCCP 16006, and NCCP16011), while the other STs had only 1 strain each (NCCP 15996, ST451; NCCP 15989, ST208; NCCP 15992, ST358; NCCP 15994, ST369; Lab-WT, ST112) (Table 2). Genotyping analyses of clinical A. baumannii isolates using the 147 CRAB isolates isolated in 2013 to 2015 revealed that 17 isolates (11.6%) belonged to ST357. However, the dominant isolates from 2016 to 2018 belonged to ST369 (21.8% [21/96 strains]), and ST451 (35.4% [34/96 strains]) evolved from ST208 because of mutations in the *gpi* allele. On the other hand, isolates belonging to ST357 were not detected in the Republic of Korea (32, 34). The high prevalence (55% [5/9 strains]) of ST357 in MDR isolates obtained from patients in the present study was probably associated with regional bias in clinical A. baumannii isolates due to nosocomial infections; however, our small sample size was not statistically significant (35). Interestingly, only two genes (*gyrB* and *gpi*) were featured as allelic variants, and the other five genes showed the same allele numbers in all of the MDR strains (Table 2). Evolutionary analyses based on the allelic profile demonstrated that three STs (ST358, ST369, and ST451) were distinct from ST208 because of a mutation in *gpi* and that ST357 had evolved from ST358 because of a single-locus mutation in the *gyrB* allele (Table 2; also see Fig. S1). The nucleotide sequence homology of *gpi* in ST369 was significantly different from that in other STs (ST208 [93%], ST358 [92%], and ST451 [95%]). Our MLST classification revealed six STs in the tested strains, suggesting that all of the MDR strains were international clone (IC) II (ST208, ST357, ST358, ST369, and ST451) except for the Lab-WT strain (IC III [ST112]) (30, 34).

**TABLE 2:** Genotyping using MLST and genetic analysis^*a*^

Strain	MLST (Oxford scheme) allele type for:							ST	IC	IS*AbaI*/*bla*_OXA-23_	*naxD*	*pmrC* homolog
	*gltA*	*gyrB*	*gdhB*	*recA*	*cpn60*	*gpi*	*rpoD*					
Reference strain												
ATCC 17978 (Lab-WT)	1	12	56	36	1	61	26	112	III	X	O	*pmrC*
PMB^R^ strains												
NCCP 16007	1	12	3	2	2	145	3	357	II	O	O	*pmrC*, *eptA-1*
NCCP 15996	1	3	3	2	2	142	3	451	II	O	O	*pmrC*, *eptA-1*
NCCP 15995	1	12	3	2	2	145	3	357	II	O	O	*pmrC*, *eptA-1*
F-1629	1	12	3	2	2	145	3	357	II	O	O	*pmrC*, *eptA-1*
PMB^S^ strains												
NCCP 15989	1	3	3	2	2	97	3	208	II	O	O	*pmrC*, *eptA-1*
NCCP 16006	1	12	3	2	2	145	3	357	II	O	O	*pmrC*, *eptA-1*
NCCP 16011	1	12	3	2	2	145	3	357	II	O	O	*pmrC*, *eptA-1*
NCCP 15992	1	3	3	2	2	145	3	358	II	O	O	*pmrC*, *eptA-1*
NCCP 15994	1	3	3	2	2	106	3	369	II	O	O	*pmrC*, *eptA-1*

a Six STs were identified on the basis of MLST following the Oxford scheme. IS*AbaI*/*bla*_OXA-23_ encoding carbapenemase and the *pmrC* homolog *eptA-1* were detected in the MDR strains. *gltA*, citrate synthase; *gyrB*, DNA gyrase subunit B; *gdhB*, glucose dehydrogenase B; *recA*, homologous recombination factor; *cpn60*, 60-kDa chaperonin; *gpi*, glucose-6-phosphate isomerase; *rpoD*, RNA polymerase sigma factor. O, present in the genome; X, absence in the genome.

**FIG 1:**
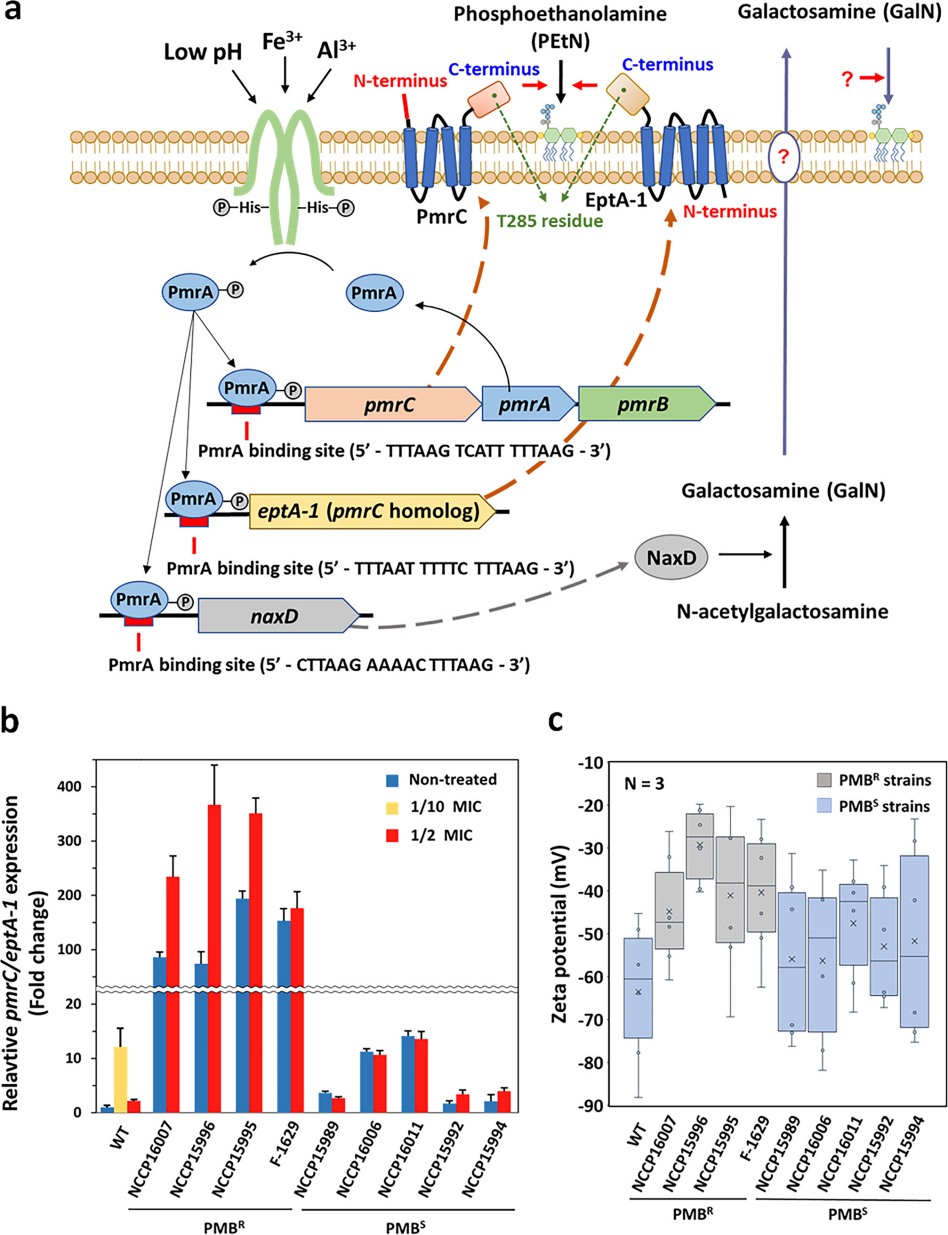
PmrAB-mediated *pmrC*/*eptA-1* expression and cell surface charge. (a) Schematic diagram of PmrAB-induced *pmrC*/*eptA-1* expression. Mutated PmrB continuously activates PmrA, and the resulting activated PmrA regulator binds to the PmrA-binding site (5′-HTTAAD-N_5_-HTTAAD-3′) to promote the transcription of *pmrC* and *eptA-1*. (b) Expression levels of *pmrC*/*eptA-1* encoding PEtN transferase were examined in clinical strains and compared with those in the Lab-WT strain under the nontreated, 1/2 MIC PMB-treated, and 1/10 MIC PMB-treated conditions. The expression of the strains was normalized to their respective 16S rRNA expression levels. Data were obtained through three independent biological replicates. (c) Zeta potential of the Lab-WT, PMB^R^, and PMB^S^ strains under the nontreated condition.

The *bla*_OXA-23_ gene, encoding a carbapenemase (GenBank accession number AY795964), is abundant in many clinical A. baumannii strains belonging to IC II worldwide; this was also noted in our tested strains (36). Genetic analyses of 160 clinical A. baumannii isolates from the Republic of Korea in 2016 to 2017 revealed that over 96% of CRAB isolates harbored *bla*_OXA-23_ with IS*AbaI* (37). Along with the discovery of the IS*AbaI*/*bla*_OXA-23_ cassette in the draft genomes of all of the tested MDR strains, it is worth noting that our MDR strains were experimentally and genetically proven to be CRAB strains (Tables 1 and 2). Interestingly, a bidirectional transcription system, P_in_ and P_out_, is predicted to be inside the structural gene encoding an IS*AbaI*-containing transposase, which may trigger the expression of *bla*_OXA-23_ (see Fig. S2). Additional *eptA* was detected in the draft genome analyses and showed 85% (1,362/1,602 bp) nucleotide sequence identity to *pmrC*. Domain analyses of both PmrC and EptA-1 proteins revealed that they had different numbers of transmembrane (TM) domains (four and five TM domains, respectively) and that a signal peptide was present only in PmrC (Fig. 1a). Given their high amino acid identity (93%) and the same enzymatic function as a PEtN transferase, they likely evolved after gene duplication, and an increased gene dosage could confer high levels of PMB resistance to cells (24, 38). In addition, the C terminus of both enzymes possessing PEtN transferase activity (highly conserved T285) is located on the periplasmic side of the IM and catalyzes the addition of PEtN to hepta-acylated lipid A (Fig. 1a) (39). Draft genome analysis demonstrated that extra *eptA-1* was also present in all of the tested MDR strains (but not in the Lab-WT strain), and their nucleotide sequences showed 100% identity to known *eptA-1* (GenBank accession number KC700024) in A. baumannii strains (Table 2) (24). A PmrA-binding motif (5′-HTTAAD-N_5_-HTTAAD-3′) was detected upstream of the promoter regions of the *pmrCAB* operon (5′-TTTAAG-TCATT-TTTAAG-3′), *eptA-1* (5′-TTTAAT-TTTTC-TTTAAG-3′), and *naxD* (5′-CTTAAG-AAAAC-TTTAAG-3′) (Fig. 1a and Table 2). PmrA-induced NaxD is known to deacetylate *N*-acetylgalactosamine, forming GalN in the cytoplasm. However, additional enzymes for the transport and attachment of GalN to lipid A have not yet been identified (40).

### Induction of *pmrC*/*eptA-1* expression and alteration of membrane charge in PMB^R^ strains.

High basal expression levels of *pmrC*/*eptA-1 c*ould contribute to high PMB resistance in the PMB^R^ strains (MIC, ~128 to 256 μg/mL), and those genes were still inducible by PMB (1/2 MIC) (Fig. 1b). The same PMB concentration (1/2 MIC) could not upregulate *pmrC*/*eptA-1* in the PMB^S^ strains, probably because of their vulnerability under high concentrations of PMB, which was also observed in the Lab-WT strain (Fig. 1b). Low concentrations of PMB (1/10 MIC) and not high concentrations (1/2 MIC) could upregulate the target genes in the Lab-WT strain, implying that exposure to high PMB concentrations made it too challenging for cells to perform normal gene transcription and metabolic activities.

In the absence of PMB, the basal expression level of *pmrC*/*eptA-1* encoding a PEtN transferase in the PMB^R^ strains (74- to 194-fold) was significantly higher than that in the PMB^S^ strains (1.4- to 14.1-fold) and the Lab-WT strain (Fig. 1b) (24, 25). Higher expression levels of PmrAB TCS-regulated and nonregulated *pmrC* led to higher PEtN transferase activity, resulting in the accumulation of PMB^R^ lipid A-PEtN instead of canonical lipid A in the OM.

**FIG 2:**
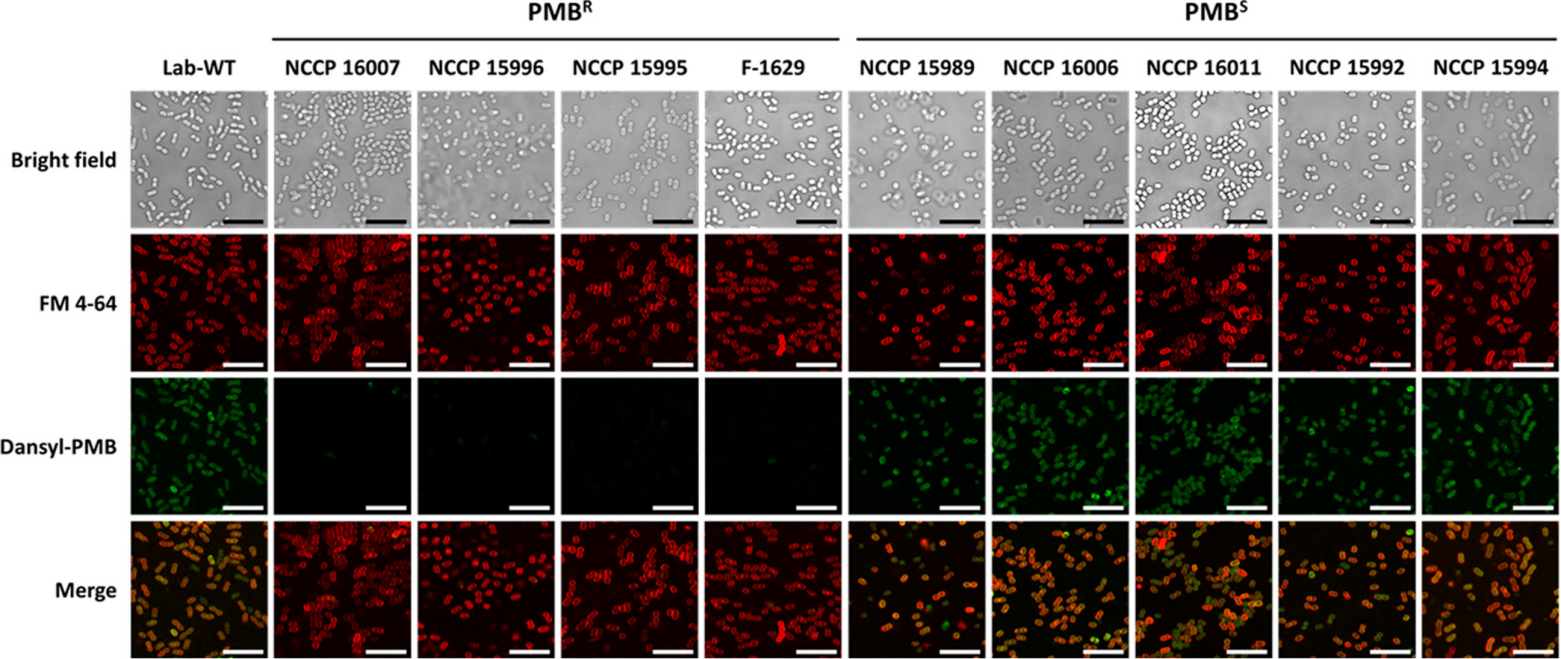
Dansyl-PMB binding affinity in Lab-WT and clinical isolates. CLSM images of Lab-WT, PMB^R^, and PMB^S^ strains are shown. The fluorescence of the lipophilic dye FM 4-64 (red) was observed in all of the strains, indicating that the membrane was well maintained. The fluorescence of dansyl-PMB (green) was observed in the Lab-WT and PMB^S^ strains but not in the PMB^R^ strains. The FM 4-64 and dansyl-PMB images were merged. Scale bars represent 10 μm.

Possible changes in the cell surface charge with the incorporation of PEtN into lipid A were monitored by measuring the zeta potential (Fig. 1c). The four PMB^R^ strains showed a lower average negative surface charge (−22 to −53 mV) than the five PMB^S^ strains (−32 to −74 mV) at the early exponential phase (optical density at 600 nm [OD_600_] of 0.4), possibly resulting in reduced initial binding of cationic PMB to the cell surface (Fig. 1c). The net negative charge of the cell surface in the PMB^R^ strains could be diminished through the addition of PEtN to lipid A by higher PEtN transferase activity. The decreased binding of PMB to the cell surface could also be confirmed using fluorescent dansyl-PMB (Fig. 2) (41). The lipophilic dye FM 4-64 is an amphipathic molecule with a lipophilic tail and trivalent cation moiety, which can interact with LPS by both electrostatic and hydrophobic interactions (42). The addition of FM 4-64 to cells could result in homogeneous fluorescence across the cell surface in all PMB^S^ and PMB^R^ strains, implying that the PEtN-driven modification of lipid A was not associated with hydrophobic interactions with FM 4-64 (Fig. 2) (43). However, fluorescence intensity following the addition of dansyl-PMB could be detected in the PMB^R^ strains but not in the PMB^S^ strains, indicating that the lower initial binding of PMB rendered PMB resistance in the PMB^R^ cells.

### Modification of lipid A in PMB^R^ strains.

**FIG 3:**
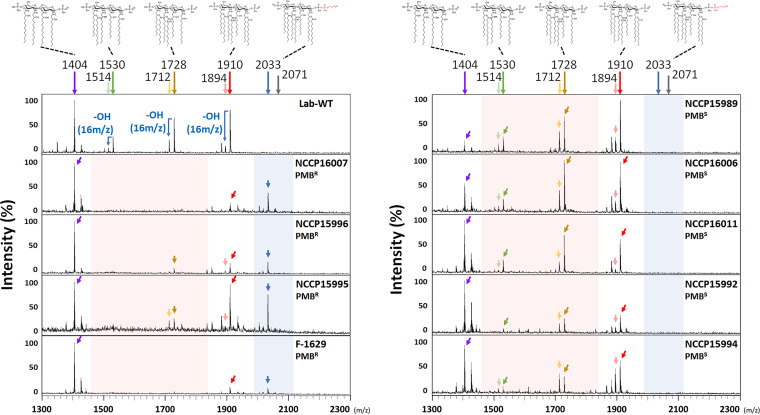
MS analysis of clinical isolates. Extracted lipid A was analyzed by MALDI-TOF MS to assess the lipid A pattern corresponding to PMB resistance. The *m/z* 2,033 peak (blue arrows) corresponding to lipid A with added PEtN was specifically detected in the PMB^R^ strains, and the *m/z* 1,530 and *m/z* 1,728 peaks (red arrows) corresponding to deacylated lipid A were detected to a lesser extent or not detected.

MS analyses of lipid A revealed significantly different patterns between the PMB^R^ and PMB^S^ strains (Fig. 3). The following four major peaks were detected in all of the tested strains, including the reference Lab-WT strain (ATCC 17978): tetra-acylated lipid A precursor (*m/z* 1,404); hepta-acylated lipid A (*m/z* 1,910), the common form in A. baumannii; hexa-acylated lipid A (*m/z* 1,728) lacking one laurate (C_12_); and penta-acylated lipid A (*m/z* 1,530) lacking two laurate molecules (C_12_) with one hydroxyl group (Fig. 3) (44). The predominant *m/z* 1,404 peak in most isolates represented a precursor located in the cytoplasmic leaflet of the IM at the early stages of lipid A biosynthesis, indicating that the extraction of lipid A could contain all lipid A precursors and complete forms on both the IM and OM sides (see Fig. S3a). The *m/z* 1,910 peak (hepta-acylated lipid A) represented the canonical lipid A of A. baumannii and was also detected in other clinical Acinetobacter strains, such as Acinetobacter pittii and Acinetobacter nosocomialis (45). PMB^R^ A. baumannii strains having lipid A components modified with PEtN (+*m/z* 123) or GalN (+*m/z* 161) at one or both terminal phosphate residues have been often reported (44). The *m/z* 2,033 peak specific to the PMB^R^ strains corresponding to lipid A and one PEtN residue (+*m/z* 123) represented hepta-acylated lipid A (lipid A-PEtN, *m/z* 1,910) (Fig. 3). However, the *m/z* 2,071 peak corresponding to the NaxD-mediated GalN modification of lipid A was not detected in the mass spectrum of lipid A, indicating that GalN-mediated lipid A modification may be a minor process for PMB resistance in the PMB^R^ strains (24). An LpxO homolog present in the genomes of S. enterica, K. pneumoniae, and A. baumannii functions as a hydroxylase working on laurate (C_12_) linked to the 2′-*R*-3-hydroxymyristoyl position of lipid A (46). The weakly detected subvariant lipid A precursors having a lower mass (−*m/z* 16 [one hydroxyl group]) than the three major peaks (*m/z* 1,910, *m/z* 1,728, and *m/z* 1,530) were detected mainly in the PMB^S^ strains but not in most PMB^R^ strains. The identification of many hydroxylated or nonhydroxylated acyl chains of lipid A predominantly in the PMB^S^ strains implied different relative speeds and degrees of cognate enzymatic activities in the strains. Interestingly, the occurrence of PEtN addition only to hepta-acylated lipid A proved the presence of PEtN activity in the periplasmic leaflet of the IM in the PMB^R^ strains. Different levels of expression of genes involved in lipid A biosynthesis (*lpxC*, *lpxK*, *lpxL*, *lpxM*, and *lptC*) occurred in the PMB^S^ and PMB^R^ strains. It is difficult to draw any conclusion regarding the presence of multiple precursors from lipid A analyses based on the level of gene expression; however, it is worth noting that the rate-limiting steps present in lipid A biosynthesis may generate different amounts of lipid A precursors in the strains (see Fig. S3a [red-marked lines] and b).

### PmrCAB variation in PMB^R^ strains.

The amino acid alterations in PmrCAB in the clinical strains were analyzed and compared with that in the Lab-WT strain, which could explain their target gene expression and constative PMB^R^ phenotypes, because mutations accumulated in the PmrAB TCS can lead to the activation of PmrA, a transcriptional regulator of downstream genes (see Table S3). Sequence analysis of the PmrB kinase of PmrAB TCS (A1S_2754) revealed that the four PMB^R^ strains with high PMB MICs had interesting and novel amino acid mutations and deletions (F26L mutation and deletion of amino acids at positions 27 to 30 in NCCP 16007; A138T mutation in NCCP 15996; and S61G and I163N mutations in NCCP 15995). In clinical strains, a mutation in PmrB (P233S, T235N, or Q270P) appeared to be important for the constitutive activation of PmrA; however, the same mutation could not be detected in our tested clinical strains (47). The P170L mutation in PmrB has been reported in PMB^R^ clinical A. baumannii strains, and the same mutation was detected in the F-1629 strain in our study (48). The kinase domain of PmrB is known to be activated by a conformational change in the sensor domain in response to environmental stimuli, such as low pH and high Fe^3+^ or Al^3+^ (49). Our analysis revealed that a mutation (P170L) in the kinase domain of PmrB occurred in the F-1629 strain. Four mutations and the deletion of PmrB in the vicinity of the periplasmic sensor domain (F26L, S61G, A138T, and I163N mutations and deletion of amino acids at positions 27 to 30) may activate the kinase domain without environmental stimuli; however, their contribution to constitutive *pmrC* expression has not yet been experimentally proven and remains to be investigated (see Fig. S4). Interestingly, mutations in PmrB were observed only in the PMB^R^ strains. No mutation was detected in PmrA (A1S_2753), and five amino acid mutations (F150L, I212V, R332K, A354S, and K515T) in PmrC (A1S_2752) were identical in all of the clinical strains.

## DISCUSSION

Our MIC tests and draft genome analysis indicated that the selected 9 clinical isolates (among the 40 tested strains) were MDR strains that possessed multiple ARGs, which could confer high levels of resistance to meropenem and other antibiotics (Table 1). In addition, our draft genomic data indicated the presence of the IS*AbaI*/*bla*_OXA-23_ cassette, *pmrC*, and *eptA*-1 in all of the tested MDR strains (Table 2; also see Fig. S2 in the supplemental material). The three major STs corresponding to IC II (ST208, ST357, and ST369) are known to contain *bla*_OXA-23_, with more than 60% prevalence in the Republic of Korea; this is consistent with our finding that all of our tested strains belonged to IC II (Table 2) (24). In the case of E. coli, the OM retained 70% of the lipid A fraction, and 23% reduction in the OM charge occurred with the addition of PEtN, which explained our zeta potential data on lowering cationic PMB affinity by reducing the net negative charge of cell surfaces in the PMB^R^ strains (Fig. 1c) (50). The addition of PEtN to lipids could also stabilize the membrane by increasing the intermolecular attraction between adjacent lipids (51). Lipid A modification in PMB^R^ A. baumannii is known to occur by the addition of PEtN or GalN, which can neutralize and stabilize membranes (52). PEtN modification mainly occurs at the C1′ position of lipid A, and a change in the C4′ position occurs with low frequency (44, 45). The addition of GalN is associated only with the C1′ position, which may explain the rare detection of GalN-modified lipid A because of positional competition with the PEtN moiety. Moreover, in our study, no production of GalN-lipid A was detected (mass peak at *m/z* 2,071), even though *naxD*, which was first discovered in a Francisella novicida clinical isolate, was present in the A. baumannii genome (Fig. 1a and Fig. 3) (44, 53). The mechanism underlying the transfer of GalN to lipid A has not yet been identified.

Interestingly, a phenomenon of LPS loss was noted in laboratory-evolved PMB^R^ A. baumannii strains when mutations occurred in *lpxA*, *lpxC*, and *lpxD* (25). The LPS-deficient cells appeared to have high PMB resistance and high sensitivity to antibacterial lysozyme and lactoferrin. Our MS data revealed only the *m/z* 1,910 peak corresponding to canonical lipid A (Fig. 3). Mutations in PhoQ, a histidine kinase in P. aeruginosa, S. enterica, and K. pneumoniae, are also attributed to lipid A modification through induction of the activation of the *arnBCADTEF* operon or the *phoPQ*-*pmrD*-*pmrAB* signal transduction cascade as a result of uncontrolled TCS functioning (6). However, A. baumannii lacks the *pmrD* and *arnBCADTEF* operons. Consequently, conferring PMB resistance to A. baumannii cells through charge modification of lipid A mainly relies on the PmrAB TCS, with the exception of LPS deficiency (54). The high basal expression levels of *pmrC*/*eptA-1* (74- to 194-fold) noted only in the PMB^R^ strains suggested that the *pmrAB* TCS was no longer strictly controlled inside the cells, possibly because of mutations accumulated in PmrB, a kinase in the PmrAB TCS. However, the direct contribution of those mutations to high basal expression levels of *pmrC* remains to be investigated (Fig. 1b; also see Table S3). Conformational changes caused by mutations in the sensor and kinase domains of PmrB can lead to a constantly activated state of PmrB. Continuous exposure of the sensory protein PmrB to environmental stimuli, such as positive ions, metals, pH, PMB, and other cationic peptides, may result in spontaneous mutation and protein evolution under selection pressure in hostile host environments (19, 25, 41). Amino acid analyses of PmrCAB in MDR strains revealed mutations in the PMB^R^ strains at amino acid positions 26 to 170 of PmrB, indicating a possible conformational change in PmrB in the PMB^R^ strains (see Fig. S4 and Table S3). In many clinical isolates, such as E. coli, K. pneumoniae, P. aeruginosa, and A. baumannii strains, PmrB mutations were found to occur at various amino acid positions, resulting in high PMB resistance (8 to 512 μg/mL) (15). In conclusion, all of the tested clinical A. baumannii isolates were proven to be MDR strains with resistance to different classes of antibiotics, but their degree of PMB resistance differed. The four selected PMB^R^ strains had mutations in PmrB, high basal expression levels of *pmrC*/*eptA-1*, and PEtN-modified lipid A without exposure to PMB (Fig. 3). These mutations and constitutive phenotypic changes confer high PMB resistance to the PMB^R^ strains. Consequently, spontaneous mutations and acquired ARGs during their residence inside hosts could contribute to their successful survival as pathogens in clinical settings.

## MATERIALS AND METHODS

### Acquisition of strains and measurement of their MICs.

Five clinical isolates of A. baumannii from a patient were provided by the Samsung Medical Center, Sungkyunkwan University, while 35 other clinical isolates were provided by the National Culture Collection for Pathogens (NCCP) in the Republic of Korea. These clinical isolates were deposited from 2004 to 2013 (30). A. baumannii ATCC 17978 (Lab-WT) was used as the reference strain; it was maintained in the laboratory after being provided by the American Type Culture Collection (ATCC). Antibiotic susceptibility tests were performed using the broth dilution method in 96-well plates to identify MDR A. baumannii strains (30). All antibiotics were purchased from Sigma-Aldrich (USA), and the following concentrations were tested: PMB, 1 to 256 μg/mL; meropenem, 0.5 to 64 μg/mL; doxycycline, 0.5 to 64 μg/mL; gentamicin, 0.5 to 512 μg/mL; erythromycin, 0.5 to 512 μg/mL. All isolates were cultured overnight in Luria-Bertani (LB) broth and then diluted (1:100) into fresh LB broth (5 mL) and incubated until they reached the early exponential phase (OD_600_ of 0.4). The cultured cells were transferred into a 96-well plate with each antibiotic, and the final cell numbers were adjusted (~10^6^ CFU/mL). Incubation was continued at 37°C for 24 h to calculate the MIC. The antibiotic resistance criteria for each antibiotic were determined using EUCAST clinical breakpoints v.12.0 for Acinetobacter species (updated in 2022) (https://www.eucast.org/fileadmin/src/media/PDFs/EUCAST_files/Breakpoint_tables/v_12.0_Breakpoint_Tables.pdf), as follows: PMB, 3 μg/mL; meropenem, 2 μg/mL; doxycycline, 2 μg/mL; gentamicin, 5 μg/mL; erythromycin, 16 μg/mL.

### Extraction of genomic DNA.

Genomic DNAs were extracted from all of the tested A. baumannii strains using the Wizard genomic DNA purification kit (Promega, USA), according to the manufacturer’s instructions. The cell pellet was obtained by centrifugation at 12,000 × *g* for 1 min using A. baumannii that had been cultured overnight, and the supernatant was discarded. The pellet was resuspended in nuclei lysis solution (600 μL) and incubated at 80°C for 5 min to lyse the cells. To remove RNAs, RNase solution (3 μL) was added to the cell lysate and incubated at 37°C for 30 min. The supernatant containing DNA was transferred into a new microtube containing isopropanol (600 μL), mixed by gentle inversion of the tube, and centrifuged at 12,000 × *g* for 2 min. The supernatant was poured out, and 70% ethanol (600 μL) was added to wash the DNA pellets. Following this, DNA rehydration solution (100 μL) was added to the DNA pellet, and the DNA was dehydrated by incubation at 65°C for 1 h. The concentration of extracted genomic DNA was assessed using a NanoPhotometer N50 (Implen, Germany), and the DNA was used for MLST and nucleotide analysis of *pmrCAB*.

### Genotypic analysis using MLST.

Extracted genomic DNA was used to perform MLST according to the Oxford scheme, as described previously, using primers designed for PCR amplification and sequencing in the PubMLST database (https://pubmlst.org/organisms/acinetobacter-baumannii) (see Table S1 in the supplemental material) (33). EzPCR HF 5× master mix (Elpis Biotech, Republic of Korea) containing a *Pfu* polymerase was used to ensure a low error rate and accurate PCRs under the following conditions: 95°C for 5 min; 30 cycles at 95°C for 30 s, 55°C for 30 s, and 72°C for 30 s; and a final cycle at 72°C for 5 min. PCR products were separated in a 1% agarose gel and purified from the gel using Expin Combo GP (GeneAll, Republic of Korea). The purified PCR products were analyzed by the Sanger sequencing method (Macrogen, Republic of Korea) using primers according to the application (see Table S1). The sequence data sets were assigned to the Acinetobacter MLST database (http://pubmlst.org/abaumannii [accessed 25 June 2021]). Draft genome sequences were obtained using the NovaSeq 6000 platform (Illumina).

### Relative quantification of *pmrC* and *eptA-1* expression.

The expression levels of *pmrC* and *eptA-1* (*pmrC*/*eptA-1*) were assessed in the presence or absence of PMB. Primers were designed from the common sequence of the conserved regions of *pmrC* and *eptA-1*, and the integrated expression levels of both genes were measured simultaneously (see Table S1). All of the clinical isolates were diluted (1:100) using overnight cultures in LB broth (5 mL) and were grown to the early exponential phase (OD_600_ of 0.4). For the PMB treatment condition, all of the strains were diluted (1:100) using overnight cultures in LB broth (5 mL) and were grown to the early exponential phase (OD_600_ of 0.4). Following this, 1/2 MIC (1 to 128 μg/mL) of PMB was added and additionally incubated for 30 min. Total RNA from the cultured cells was isolated using the RNeasy minikit (Qiagen, Germany), according to the manufacturer’s protocol. Reverse transcription was performed using the RevertAid reverse transcription kit (Thermo Fisher Scientific, USA) with DNase I (1 μL [Thermo Fisher Scientific])-treated RNA. Quantitative real-time PCR (qRT-PCR) amplification was performed on the QuantStudio 5 real-time PCR system (Applied Biosystems, USA) using Power SYBR green PCR master mix (Applied Biosystems). For RT-PCR, the total mixture (20 μL) was prepared as follows: master mix (10 μL), forward primer (1 μL [10 pmol]), reverse primer (1 μL [10 pmol]), diluted (1/10) cDNA (1 μL) as a template, and distilled water (7 μL). qRT-PCR was performed under the following conditions: hold stage, 95°C for 10 min; PCR stage, 40 cycles at 95°C for 15 s and 60°C for 1 min; melt curve stage, 95°C for 15 s, 60°C for 1 min, and 95°C for 15 s. The relative expression level of *pmrC*/*eptA-1* was normalized with 16S rRNA as an endogenous gene. The primers used are listed in Table S1 in the supplemental material.

### Measurement of zeta potential.

The cell surface charge was measured using a zeta potential analyzer, as described previously (41). Cells cultured overnight were diluted (1:100) into fresh LB broth (5 mL) and grown to the early exponential phase (OD_600_ of 0.4). To wash the residual ions on the cell surface, the cultured cells were centrifuged at 12,000 × *g* for 1 min and washed with phosphate-buffered saline (PBS) (pH 6.8) twice. The washed pellet was resuspended in PBS and, immediately before measurement of the zeta potential, was diluted one to three times in PBS according to the cell density. The zeta potential of the bacterial cells was measured at 25°C using the ELSZ-1000 zeta potential analyzer (Otsuka Electronics, Japan).

### Image analysis using confocal laser scanning microscopy.

Dansyl-PMB was prepared using fractions from column chromatography after combining dansyl chloride (Sigma-Aldrich) with PMB sulfate (Sigma-Aldrich), as described previously (41). The lipophilic dye FM 4-64 [*N*-(3-triethylammoniumpropyl)-4-(6-[4-(diethylamino)phenyl]hexatrienyl) pyridinium dibromide] (Invitrogen, USA) reveals the cell membrane by attaching to the lipid of the OM. Cells in the early exponential phase (OD_600_ of 0.4) (1 mL) were centrifuged in a microtube. The pellet was resuspended in 1 mL of PBS. Fluorescently labeled dansyl-PMB (final concentration, 2 μg/mL) and the lipophilic dye FM 4-64 (final concentration, 0.1 μg/mL) were added for cell staining and incubated for 30 min at 37°C. The treated cells were washed with PBS more than twice. The washed pellet was prepared by resuspension in PBS (10 μL). Confocal laser scanning microscopy (CLSM) was performed using an LSM 700 microscope (Carl Zeiss Microscopy, Germany).

### Microextraction of lipid A.

Lipid A of all of the tested strains was extracted using the modified ammonium hydroxide-isobutyric acid method (25). In brief, cells cultured overnight were diluted (1:100) into fresh LB broth (400 mL). The culture was then incubated with vigorous shaking at 37°C until the OD_600_ reached 1.0. Bacterial pellets were obtained from the cultured cells by high-speed centrifugation (Hitachi, Japan) at 4,000 × *g* for 20 min at 4°C. The supernatant was poured out, and the pellets were washed with distilled water (30 mL) twice to remove residual ions. The washed cell pellets were snap-frozen using liquid nitrogen and lyophilized overnight. Lyophilized crude cells (10 mg) were suspended in a mixture of isobutyric acid and 1 M ammonium hydroxide (400 μL, 5:3 [vol/vol]; Sigma-Aldrich) and incubated for 2 h at 100°C in a screw-cap test tube with occasional vortex-mixing. The mixture was then cooled on ice and centrifuged at 8,000 × *g* for 15 min. The supernatant was transferred into a new tube, mixed with an equal volume of water, and snap-frozen using liquid nitrogen before being lyophilized overnight. The lyophilized sample was then washed twice with methanol (400 μL) and centrifuged at 5,000 × *g* for 15 min. Finally, insoluble lipid A was solubilized in a chloroform-methanol-water mixture (100 μL, 3:1.5:0.25 [vol/vol/vol]). The extract was directly subjected to MALDI-TOF MS analysis.

### MS analysis.

Lipid A extracted from whole cells using the microextraction method was subjected to MALDI-TOF MS analysis (25). In brief, 2,5-dihydroxybenzoic acid (DHB) matrix solution (10 mg/mL in 80% acetonitrile with 0.1% trifluoroacetic acid) was used for MS. Lipid A extract (2 μL) was mixed with matrix solution (2 μL) deposited on a plate for the vacuum-dried droplet method. The structural spectrum of lipid A was analyzed using the autoflex maX mass spectrometer (Bruker Daltonics, USA) in the negative-ion reflectron mode. The laser repetition rate was 2,000 Hz, and the resulting spectrum was accumulated with an average of 500 shots. The total analysis range of MALDI-TOF MS was *m/z* 400 to *m/z* 3,200, and lipid A mass analysis was performed at *m/z* 1,300 to *m/z* 2,300.

### Protein sequence analysis of PmrCAB in clinical isolates and the Lab-WT isolate.

To determine whether amino acid substitution contributes to PMB resistance, the *pmrCAB* operon was amplified using a *Pfu* polymerase with proofreading activity. Based on the reference genome sequence of the Lab-WT strain (GenBank accession number CP000521), three sets of PCR primers were designed to encompass the entire *pmrCAB* operon along with the promoter regions containing the PmrA binding site in the promoter region (see Table S1). EzPCR HF 5× master mix (Elpis Biotech, Republic of Korea) was used, and three fragments were amplified using three pairs of primers, according to the manufacturer’s protocol (see Table S1). PCRs were performed under the following conditions: 95°C for 5 min; 30 cycles at 95°C for 30 s, 55°C for 30 s, and 72°C for 1 min 30 s; and a final cycle at 72°C for 5 min. Sequenced fragments were assembled and aligned to the *pmrCAB* sequence of the reference genome of the Lab-WT strain (GenBank accession number CP000521). The functional domain model of each gene was predicted using the Web-based programs InterPro (https://www.ebi.ac.uk/interpro) and Protter (http://wlab.ethz.ch/protter/start).

### Data availability.

We confirm that the data supporting the findings of this study are available within the article and/or its supplemental material. The genomic data for eight MDR strains were deposited in the NCBI GenBank database under the following GenBank accession numbers: A. baumannii NCCP 15989, JAKVTK000000000; NCCP 15992, JAKVTL000000000; NCCP 15994, JAKVTM000000000; NCCP 15995, JAKVTN000000000; NCCP 15996, JAKVTO000000000; NCCP 16006, JAKVTP000000000; NCCP 16011, JAKVTQ000000000; F-1629, JAKVTJ000000000. The complete genome sequence of A. baumannii NCCP 16007 (GenBank accession number CP091465) was obtained by constructing a hybrid genome using Pacific Biosciences (PacBio) and Illumina data and was deposited in the NCBI GenBank database. In addition, A. baumannii ATCC 17978 (GenBank accession number CP000521) was used as the reference strain.

## References

[1] Simpson BW, Trent MS. 2019. Pushing the envelope: LPS modifications and their consequences. Nat Rev Microbiol 17:403–416. doi:10.1038/s41579-019-0201-x. 31142822 PMC6913091

[2] Harding CM, Hennon SW, Feldman MF. 2018. Uncovering the mechanisms of *Acinetobacter baumannii* virulence. Nat Rev Microbiol 16:91–102. doi:10.1038/nrmicro.2017.148. 29249812 PMC6571207

[3] Jeon J, Park JH, Yong D. 2019. Efficacy of bacteriophage treatment against carbapenem-resistant *Acinetobacter baumannii* in *Galleria mellonella* larvae and a mouse model of acute pneumonia. BMC Microbiol 19:70. doi:10.1186/s12866-019-1443-5. 30940074 PMC6444642

[4] Strateva T, Sirakov I, Stoeva T, Stratev A, Dimov S, Savov E, Mitov I. 2019. Carbapenem-resistant *Acinetobacter baumannii*: current status of the problem in four Bulgarian university hospitals (2014–2016). J Glob Antimicrob Resist 16:266–273. doi:10.1016/j.jgar.2018.10.027. 30412782

[5] Han ML, Zhu Y, Creek DJ, Lin YW, Anderson D, Shen HH, Tsuji B, Gutu AD, Moskowitz SM, Velkov T, Li J. 2018. Alterations of metabolic and lipid profiles in polymyxin-resistant *Pseudomonas aeruginosa*. Antimicrob Agents Chemother 62:e02656-17. doi:10.1128/AAC.02656-17. 29632014 PMC5971563

[6] Trimble MJ, Mlynárčik P, Kolář M, Hancock RE. 2016. Polymyxin: alternative mechanisms of action and resistance. Cold Spring Harb Perspect Med 6:a025288. doi:10.1101/cshperspect.a025288. 27503996 PMC5046685

[7] Steimle A, Autenrieth IB, Frick JS. 2016. Structure and function: lipid A modifications in commensals and pathogens. Int J Med Microbiol 306:290–301. doi:10.1016/j.ijmm.2016.03.001. 27009633

[8] Raetz CR, Reynolds CM, Trent MS, Bishop RE. 2007. Lipid A modification systems in Gram-negative bacteria. Annu Rev Biochem 76:295–329. doi:10.1146/annurev.biochem.76.010307.145803. 17362200 PMC2569861

[9] Paracini N, Clifton LA, Skoda M, Lakey JH. 2018. Liquid crystalline bacterial outer membranes are critical for antibiotic susceptibility. Proc Natl Acad Sci USA 115:E7587–E7594. doi:10.1073/pnas.1803975115. 30037998 PMC6094139

[10] Vanni S, Riccardi L, Palermo G, De Vivo M. 2019. Structure and dynamics of the acyl chains in the membrane trafficking and enzymatic processing of lipids. Acc Chem Res 52:3087–3096. doi:10.1021/acs.accounts.9b00134. 31364837

[11] Sabnis A, Hagart KL, Klöckner A, Becce M, Evans LE, Furniss R, Mavridou DA, Murphy R, Stevens MM, Davies JC, Larrouy-Maumus GJ, Clarke TB, Edwards AM. 2021. Colistin kills bacteria by targeting lipopolysaccharide in the cytoplasmic membrane. eLife 10:e65836. doi:10.7554/eLife.65836. 33821795 PMC8096433

[12] Reinés M, Llobet E, Dahlström KM, Pérez-Gutiérrez C, Llompart CM, Torrecabota N, Salminen TA, Bengoechea JA. 2012. Deciphering the acylation pattern of *Yersinia enterocolitica* lipid A. PLoS Pathog 8:e1002978. doi:10.1371/journal.ppat.1002978. 23133372 PMC3486919

[13] Han ML, Velkov T, Zhu Y, Roberts KD, Le Brun AP, Chow SH, Gutu AD, Moskowitz SM, Shen HH, Li J. 2018. Polymyxin-induced lipid A deacylation in *Pseudomonas aeruginosa* perturbs polymyxin penetration and confers high-level resistance. ACS Chem Biol 13:121–130. doi:10.1021/acschembio.7b00836. 29182311

[14] Herrera CM, Voss BJ, Trent MS. 2021. Homeoviscous adaptation of the *Acinetobacter baumannii* outer membrane: alteration of lipooligosaccharide structure during cold stress. mBio 12:e01295-21. doi:10.1128/mBio.01295-21. 34425709 PMC8406137

[15] Huang J, Li C, Song J, Velkov T, Wang L, Zhu Y, Li J. 2020. Regulating polymyxin resistance in Gram-negative bacteria: roles of two-component systems PhoPQ and PmrAB. Future Microbiol 15:445–459. doi:10.2217/fmb-2019-0322. 32250173 PMC7236789

[16] Groisman EA. 2016. Feedback control of two-component regulatory systems. Annu Rev Microbiol 70:103–124. doi:10.1146/annurev-micro-102215-095331. 27607549 PMC8380452

[17] Tiwari S, Jamal SB, Hassan SS, Carvalho P, Almeida S, Barh D, Ghosh P, Silva A, Castro T, Azevedo V. 2017. Two-component signal transduction systems of pathogenic bacteria as targets for antimicrobial therapy: an overview. Front Microbiol 8:1878. doi:10.3389/fmicb.2017.01878. 29067003 PMC5641358

[18] De Silva PM, Kumar A. 2019. Signal transduction proteins in *Acinetobacter baumannii*: role in antibiotic resistance, virulence, and potential as drug targets. Front Microbiol 10:49. doi:10.3389/fmicb.2019.00049. 30761101 PMC6363711

[19] Cao Q, Yang N, Wang Y, Xu C, Zhang X, Fan K, Chen F, Liang H, Zhang Y, Deng X, Feng Y, Yang CG, Wu M, Bae T, Lan L. 2020. Mutation-induced remodeling of the BfmRS two-component system in *Pseudomonas aeruginosa* clinical isolates. Sci Signal 13:eaaz1529. doi:10.1126/scisignal.aaz1529. 33144518

[20] Howden BP, Stinear TP, Allen DL, Johnson PD, Ward PB, Davies JK. 2008. Genomic analysis reveals a point mutation in the two-component sensor gene *graS* that leads to intermediate vancomycin resistance in clinical *Staphylococcus aureus*. Antimicrob Agents Chemother 52:3755–3762. doi:10.1128/AAC.01613-07. 18644967 PMC2565880

[21] Olaitan AO, Morand S, Rolain JM. 2014. Mechanisms of polymyxin resistance: acquired and intrinsic resistance in bacteria. Front Microbiol 5:643. doi:10.3389/fmicb.2014.00643. 25505462 PMC4244539

[22] Palethorpe S, Milton ME, Pesci EC, Cavanagh J. 2022. Structure of the *Acinetobacter baumannii* PmrA receiver domain and insights into clinical mutants affecting DNA binding and promoting colistin resistance. J Biochem 170:787–800. doi:10.1093/jb/mvab102. 34585233 PMC8753958

[23] Ma F, Shen C, Zheng X, Liu Y, Chen H, Zhong L, Liang Y, Liao K, Xia Y, Tian GB, Yang Y. 2019. Identification of a novel plasmid carrying *mcr-4.3* in an *Acinetobacter baumannii* strain in China. Antimicrob Agents Chemother 63:e00133-19. doi:10.1128/AAC.00133-19. 30936095 PMC6535571

[24] Trebosc V, Gartenmann S, Tötzl M, Lucchini V, Schellhorn B, Pieren M, Lociuro S, Gitzinger M, Tigges M, Bumann D, Kemmer C. 2019. Dissecting colistin resistance mechanisms in extensively drug-resistant *Acinetobacter baumannii* clinical isolates. mBio 10:e01083-19. doi:10.1128/mBio.01083-19. 31311879 PMC6635527

[25] Lee JY, Chung ES, Ko KS. 2017. Transition of colistin dependence into colistin resistance in *Acinetobacter baumannii*. Sci Rep 7:14216. doi:10.1038/s41598-017-14609-0. 29079752 PMC5660220

[26] Powers MJ, Trent MS. 2018. Expanding the paradigm for the outer membrane: *Acinetobacter baumannii* in the absence of endotoxin. Mol Microbiol 107:47–56. doi:10.1111/mmi.13872. 29114953 PMC5740007

[27] Girardello R, Visconde M, Cayô R, Figueiredo RC, Mori MA, Lincopan N, Gales AC. 2017. Diversity of polymyxin resistance mechanisms among *Acinetobacter baumannii* clinical isolates. Diagn Microbiol Infect Dis 87:37–44. doi:10.1016/j.diagmicrobio.2016.10.011. 27776788

[28] Evans DR, Griffith MP, Sundermann AJ, Shutt KA, Saul MI, Mustapha MM, Marsh JW, Cooper VS, Harrison LH, Van Tyne D. 2020. Systematic detection of horizontal gene transfer across genera among multidrug-resistant bacteria in a single hospital. eLife 9:e53886. doi:10.7554/eLife.53886. 32285801 PMC7156236

[29] Khurshid M, Rasool MH, Ashfaq UA, Aslam B, Waseem M, Ali MA, Almatroudi A, Rasheed F, Saeed M, Guo Q, Wang M. 2020. *Acinetobacter baumannii* sequence types harboring genes encoding aminoglycoside modifying enzymes and 16S rRNA methylase; a multicenter study from Pakistan. Infect Drug Resist 13:2855–2862. doi:10.2147/IDR.S260643. 32884309 PMC7443399

[30] Kim M, Park J, Park W. 2021. Genomic and phenotypic analyses of multidrug-resistant *Acinetobacter baumannii* NCCP 16007 isolated from a patient with a urinary tract infection. Virulence 12:150–164. doi:10.1080/21505594.2020.1867421. 33372826 PMC7781626

[31] Lai CC, Chen CC, Huang HL, Chuang YC, Tang HJ. 2016. The role of doxycycline in the therapy of multidrug-resistant *E. coli* - an *in vitro* study. Sci Rep 6:31964. doi:10.1038/srep31964. 27534373 PMC4989187

[32] Jun SH, Lee DE, Hwang HR, Kim N, Kim HJ, Lee YC, Kim YK, Lee JC. 2021. Clonal change of carbapenem-resistant *Acinetobacter baumannii* isolates in a Korean hospital. Infect Genet Evol 93:104935. doi:10.1016/j.meegid.2021.104935. 34029723

[33] Bartual SG, Seifert H, Hippler C, Luzon MA, Wisplinghoff H, Rodríguez-Valera F. 2005. Development of a multilocus sequence typing scheme for characterization of clinical isolates of *Acinetobacter baumannii*. J Clin Microbiol 43:4382–4390. doi:10.1128/JCM.43.9.4382-4390.2005. 16145081 PMC1234098

[34] Jeon H, Kim S, Kim MH, Kim SY, Nam D, Park SC, Park SH, Bae H, Lee HJ, Cho JH, Lee WK, Lee YC, Lee SH, Shin MS, Lee JC. 2018. Molecular epidemiology of carbapenem-resistant *Acinetobacter baumannii* isolates from a Korean hospital that carry *bla*_OXA-23_. Infect Genet Evol 58:232–236. doi:10.1016/j.meegid.2018.01.003. 29307769

[35] Seo I, Lee J, Son SY, Han K. 2014. Comparative study of different molecular methods for typing of *Acinetobacter baumannii* clinical isolates from University Hospitals. Genes Genom 36:551–558. doi:10.1007/s13258-014-0183-z.

[36] Wang D, Yan D, Hou W, Zeng X, Qi Y, Chen J. 2015. Characterization of *bla*_OxA-23_ gene regions in isolates of *Acinetobacter baumannii*. J Microbiol Immunol Infect 48:284–290. doi:10.1016/j.jmii.2014.01.007. 24675065

[37] Yoon EJ, Kim D, Lee H, Lee HS, Shin JH, Uh Y, Shin KS, Kim YA, Park YS, Shin JH, Jeong SH. 2019. Counter clinical prognoses of patients with bloodstream infections between causative *Acinetobacter baumannii* clones ST191 and ST451 belonging to the international clonal lineage II. Front Public Health 7:233. doi:10.3389/fpubh.2019.00233. 31475131 PMC6707333

[38] Sandegren L, Andersson DI. 2009. Bacterial gene amplification: implications for the evolution of antibiotic resistance. Nat Rev Microbiol 7:578–588. doi:10.1038/nrmicro2174. 19609259

[39] Son SJ, Huang R, Squire CJ, Leung I. 2019. MCR-1: a promising target for structure-based design of inhibitors to tackle polymyxin resistance. Drug Discov Today 24:206–216. doi:10.1016/j.drudis.2018.07.004. 30036574

[40] Chin CY, Gregg KA, Napier BA, Ernst RK, Weiss DS. 2015. A PmrB-regulated deacetylase required for lipid A modification and polymyxin resistance in *Acinetobacter baumannii*. Antimicrob Agents Chemother 59:7911–7914. doi:10.1128/AAC.00515-15. 26459891 PMC4649237

[41] Park J, Kim M, Shin B, Kang M, Yang J, Lee TK, Park W. 2021. A novel decoy strategy for polymyxin resistance in *Acinetobacter baumannii*. eLife 10:e66988. doi:10.7554/eLife.66988. 34180396 PMC8324293

[42] Ginez LD, Osorio A, Vázquez-Ramírez R, Arenas T, Mendoza L, Camarena L, Poggio S. 2022. Changes in fluidity of the *E. coli* outer membrane in response to temperature, divalent cations and polymyxin-B show two different mechanisms of membrane fluidity adaptation. FEBS J 289:3550–3567. doi:10.1111/febs.16358. 35038363

[43] Roberts KD, Azad MA, Wang J, Horne AS, Thompson PE, Nation RL, Velkov T, Li J. 2015. Antimicrobial activity and toxicity of the major lipopeptide components of polymyxin B and colistin: last-line antibiotics against multidrug-resistant Gram-negative bacteria. ACS Infect Dis 1:568–575. doi:10.1021/acsinfecdis.5b00085. 27525307 PMC4980087

[44] Pelletier MR, Casella LG, Jones JW, Adams MD, Zurawski DV, Hazlett KRO, Doi Y, Ernst RK. 2013. Unique structural modifications are present in the lipopolysaccharide from colistin-resistant strains of *Acinetobacter baumannii*. Antimicrob Agents Chemother 57:4831–4840. doi:10.1128/AAC.00865-13. 23877686 PMC3811424

[45] Leung LM, McElheny CL, Gardner FM, Chandler CE, Bowler SL, Mettus RT, Spychala CN, Fowler EL, Opene BNA, Myers RA, Goodlett DR, Doi Y, Ernst RK. 2019. A prospective study of *Acinetobacter baumannii* complex isolates and colistin susceptibility monitoring by mass spectrometry of microbial membrane glycolipids. J Clin Microbiol 57:e01100-18. doi:10.1128/JCM.01100-18. 30567747 PMC6425172

[46] Bartholomew TL, Kidd TJ, Sá Pessoa J, Conde Álvarez R, Bengoechea JA. 2019. 2-Hydroxylation of *Acinetobacter baumannii* lipid A contributes to virulence. Infect Immun 87:e00066-19. doi:10.1128/IAI.00066-19. 30745327 PMC6434125

[47] Sun B, Liu H, Jiang Y, Shao L, Yang S, Chen D. 2020. New mutations involved in colistin resistance in *Acinetobacter baumannii*. mSphere 5:e00895-19. doi:10.1128/mSphere.00895-19. 32238571 PMC7113586

[48] Pournaras S, Poulou A, Dafopoulou K, Chabane YN, Kristo I, Makris D, Hardouin J, Cosette P, Tsakris A, Dé E. 2014. Growth retardation, reduced invasiveness, and impaired colistin-mediated cell death associated with colistin resistance development in *Acinetobacter baumannii*. Antimicrob Agents Chemother 58:828–832. doi:10.1128/AAC.01439-13. 24247145 PMC3910856

[49] Chen HD, Groisman EA. 2013. The biology of the PmrA/PmrB two-component system: the major regulator of lipopolysaccharide modifications. Annu Rev Microbiol 67:83–112. doi:10.1146/annurev-micro-092412-155751. 23799815 PMC8381567

[50] Khondker A, Dhaliwal AK, Saem S, Mahmood A, Fradin C, Moran-Mirabal J, Rheinstädter MC. 2019. Membrane charge and lipid packing determine polymyxin-induced membrane damage. Commun Biol 2:67. doi:10.1038/s42003-019-0297-6. 30793045 PMC6379423

[51] Khondker A, Rheinstädter MC. 2020. How do bacterial membranes resist polymyxin antibiotics? Commun Biol 3:77. doi:10.1038/s42003-020-0803-x. 32066819 PMC7026071

[52] Richie DL, Wang L, Chan H, De Pascale G, Six DA, Wei JR, Dean CR. 2018. A pathway-directed positive growth restoration assay to facilitate the discovery of lipid A and fatty acid biosynthesis inhibitors in *Acinetobacter baumannii*. PLoS One 13:e0193851. doi:10.1371/journal.pone.0193851. 29505586 PMC5837183

[53] Llewellyn AC, Zhao J, Song F, Parvathareddy J, Xu Q, Napier BA, Laroui H, Merlin D, Bina JE, Cotter PA, Miller MA, Raetz CR, Weiss DS. 2012. NaxD is a deacetylase required for lipid A modification and *Francisella* pathogenesis. Mol Microbiol 86:611–627. doi:10.1111/mmi.12004. 22966934 PMC3841722

[54] Cheah SE, Johnson MD, Zhu Y, Tsuji BT, Forrest A, Bulitta JB, Boyce JD, Nation RL, Li J. 2016. Polymyxin resistance in *Acinetobacter baumannii*: genetic mutations and transcriptomic changes in response to clinically relevant dosage regimens. Sci Rep 6:26233. doi:10.1038/srep26233. 27195897 PMC4872528

