# Supplementary material for: Constitutive Phenotypic Modification of Lipid A in Clinical Acinetobacter baumannii Isolates
Source: Microbiol Spectr. 2022 Jul 21;10(4):e01295-22. doi: 10.1128/spectrum.01295-22 (PMC9431647; doi:10.1128/spectrum.01295-22)
Supplement: Supplemental file 1 — Supplemental material. Download spectrum.01295-22-s0001.pdf, PDF file, 1.0 MB [file spectrum.01295-22-s0001.pdf]

Constitutive phenotypic modification of lipid A in clinical *Acinetobacter baumannii* isolates

Su-Hyun Kim[a], Sohyeon Yun[a], & Woojun Park[a]*

[a]*Laboratory of Molecular Environmental Microbiology, Department of Environmental Science and Ecological Engineering, Korea University, Seoul, Republic of Korea*

Keywords: Carbapenem-resistant; Polymyxin; Lipopolysaccharide; Phosphoethanolamine; *pmrC*; Gram-negative bacteria

***Corresponding author:** Dr. Woojun Park, Department of Environmental Science and Ecological Engineering, Korea University, Seoul, Republic of Korea

E-mail: wpark@korea.ac.kr

Fax: +82-2-953-0737

Phone: +82-2-3290-3067

Supplementary Table 1. Primers used in this study.

MLST primers	Sequence (5′–3′)	Reference
*gltA*_F	AATTTACAGTGGCACATTAGGTCCC	(1)
*gltA*_R	GCAGAGATACCAGCAGAGATACACG	(1)
*gyrB*_F	TGAAGGCGGCTTATCTGAGT	(1)
*gyrB*_R	GCTGGGTCTTTTTCCTGACA	(1)
*gdhB*_amp_F	GCTACTTTTATGCAACAGAGCC	(1)
*gdhB*_amp_R	GTTGAGTTGGCGTATGTTGTGC	(1)
*gdhB*_seq_F	ACCACATGCTTTGTTATG	(1)
*gdhB*_seq_R	GTTGGCGTATGTTGTGC	(1)
*recA*_F	CCTGAATCTTCYGGTAAAAC	(1)
*recA*_R	GTTTCTGGGCTGCCAAACATTAC	(1)
*cpn60*_F	GGTGCTCAACTTGTTCGTGA	(1)
*cpn60*_R	CACCGAAACCAGGAGCTTTA	(1)
*gpi*_F	GAAATTTCCGGAGCTCACAA	(1)
*gpi*_R	TCAGGAGCAATACCCCACTC	(1)
*rpoD*_F	ACCCGTGAAGGTGAAATCAG	(1)
*rpoD*_R	TTCAGCTGGAGCTTTAGCAAT	(1)
**qRT primers**	**Sequence (5′–3′)**	**Reference**
qRT_16s_F(80)	TGGTGCCTTCGGGAATCTAG	This study
qRT_16s_R(80)	TGCGGGACTTAACCCAACAT	This study
RT_*pmrC*_F(100)	TGGGTAGTCATGGACCTGCAT	This study
RT_*pmrC*_R(100)	GTTTGCGAACAGCCCTGTATC	This study
RT_*lpxC*_ F(105)	GGTGGCCTGTGTGAACAAGA	This study
RT_*lpxC*_ R(105)	GCCATTATGCGGGCTGAATA	This study
RT_*lpxK*_ F(100)	TGACGACCTAATGCCCAATG	This study
RT_*lpxK*_ R(100)	GACCCAATAGACAAGCTGCAATC	This study
RT_*lpxL*_ F(100)	ATGCACAAGCTCAAGGCAAAG	This study
RT_*lpxL*_ R(100)	ACATCCGGCTCGAAATATTGG	This study
RT_*lpxM*_ F(108)	TCCGGCAAAATTACTGTCGTT	This study
RT_*lpxM*_ R(108)	AGAAGCCCGTAGCCGTGAA	This study
RT_*lptC*_ F(105)	CGCCTTGTGGAGACTGAACTG	This study
RT_*lptC*_ R(105)	CACCGCAAGGAACCATTGA	This study

pmrCAB **operon primers**	**Sequence (5′–3′)**	**Reference**
pmr_1_F	TGGTAAGTCTTACAAAGATAACCGTGTTGGT	This study
pmr_1_R	GGTTTGCGAACAGCCCTGTATC	This study
pmr_2_F	AGATGGCGAATGTTATGATGACATTCTC	This study
pmr_2_R	CTTCTTGAAGTGCAACCTTATAAGCAC	This study
pmr_3_F	AGCGAAGCTGGGTAAAGATTTTATCC	This study
pmr_3_R	GGAGCACATTTCCTAAGCCATAAC	This study

Supplementary Table 2. Antibiotic susceptibility tests of clinical *A. baumannii* isolates used in this study. The minimum inhibitory concentrations of four classes of antibiotics were measured using the two-fold dilution method. *, MIC of polymyxin B in a previous study (2).

Strain	Origin	Year	Region	Antibiotics				
				**Polymyxin B***	**Meropenem**	**Doxycycline**	**Gentamicin**	**Erythromycin**
NCCP 12276	Other	N/A	Busan	3	2	16	>512	>512
NCCP 12277	Pus	N/A	Gwangju	1.5	≤0.5	16	>512	512
NCCP 12278	Sputum	N/A	Gyeongsangnam-do	2.5	16	0.5	32	32
NCCP 14606	Cerebrospinal fluid	2005	Daegu	2	≤0.5	≤0.5	2	4
NCCP 14607	Sputum	2005	Daegu	1	>64	32	>512	64
NCCP 14608	Pus	2004	Daegu	2	1	16	>512	>512
NCCP 14609	Pleural fluid	2009	Daegu	1	>64	1	>512	>512
NCCP 14654	Other	2009	Gyeongsangnam-do	2	64	2	>512	>512
NCCP 14655	Pus	2009	Gyeongsangnam-do	2	≤0.5	8	512	16
NCCP 14782	Pus	2009	Gyeongsangnam-do	2	≤0.5	≤0.5	2	8
NCCP 15987	Pus	2013	Incheon	2	≤0.5	≤0.5	2	16
NCCP 15988	Sputum	2013	Jeollanam-do	3	64	≤0.5	8	32
NCCP 15990	Sputum	2013	Gwangju	1.5	>64	1	>512	>512
NCCP 15991	Urine	2013	Gyeongsangbuk-do	2	64	32	>512	512

NCCP 15993	Pus	2013	Seoul	1	32	32	4	16
NCCP 15997	Urine	2013	Jeollanam-do	3	32	≤0.5	>512	>512
NCCP 15998	Sputum	2013	Gyrongsangbuk-do	2	16	16	>512	32
NCCP 15999	Sputum	2013	Gyeongsangnam-do	2.5	>64	≤0.5	>512	64
NCCP 16000	Prostate	2013	Gyeonggi-do	1.5	64	≤0.5	512	16
NCCP 16001	Other	2013	Chungcheongbuk-do	1.5	≤0.5	≤0.5	2	16
NCCP 16002	Sputum	2013	Gangwon-do	1.5	32	16	2	32
NCCP 16003	Sputum	2013	Gyeongsangnam-do	4	≤0.5	≤0.5	2	8
NCCP 16004	Urine	2013	Seoul	1	≤0.5	≤0.5	1	8
NCCP 16005	Sputum	2013	Gyeongsangnam-do	1.5	≤0.5	≤0.5	2	8
NCCP 16008	N/A	2011	Seoul	2	64	32	>512	512
NCCP 16009	N/A	2011	Seoul	1	32	32	>512	512
NCCP 16010	N/A	2011	Seoul	2	64	32	>512	512
F-1025	N/A	N/A	Seoul	4	64	1	>512	>512
F-1208	N/A	N/A	Seoul	4	64	32	>512	512
F-1379	N/A	N/A	Seoul	2	≤0.5	16	>512	64
F-1410	N/A	N/A	Seoul	2	2	32	>512	>512

Supplementary Table 3. Amino acid variations in PmrCAB in MDR clinical *A. baumannii* isolates. Common variations in *pmrC* and point mutations of distinct regions in *pmrB* were detected by genomic analysis of the *pmrCAB* operon in comparison with the reference strain (Lab-WT). PMB^R, polymyxin B-resistant strains; PMB^S, polymyxin B-susceptible strains; ▲, mutation site; △, deletion site.

Category	Isolates	PMB MIC (µg/mL)	Amino acid position															
			pmrC					*pmrA*	*pmrB*									
			150	212	332	354	515	-	26	27–30	61	138	163	170	233	376–399	400–444	444
**Reference strain**	ATCC 17978 (Lab-WT)	2	F	I	R	A	K	-	F	-	S	A	I	P	P	-	-	A
**PMB^R**	NCCP 16007	256	L	V	K	S	T	-	L	△	-	-	-	-	-	-	-	V
	NCCP 15996	256	L	V	K	S	T	-	-	-	-	T	-	-	-	-	-	V
	NCCP 15995	128	L	V	K	S	T	-	-	-	G	-	N	-	-	-	-	V
	F-1629	128	L	V	K	S	T	-	-	-	-	-	-	L	-	-	-	V
**PMB^S**	NCCP 15989	2.5	L	V	K	S	T	-	-	-	-	-	-	-	-	-	-	V
	NCCP 16006	2	L	V	K	S	T	-	-	-	-	-	-	-	-	-	-	V
	NCCP 16011	2	L	V	K	S	T	-	-	-	-	-	-	-	S	▲	△	△
	NCCP 15992	1	L	V	K	S	T	-	-	-	-	-	-	-	-	-	-	V
	NCCP 15994	1	L	V	K	S	T	-	-	-	-	-	-	-	-	-	-	V

Supplementary Figure 1.

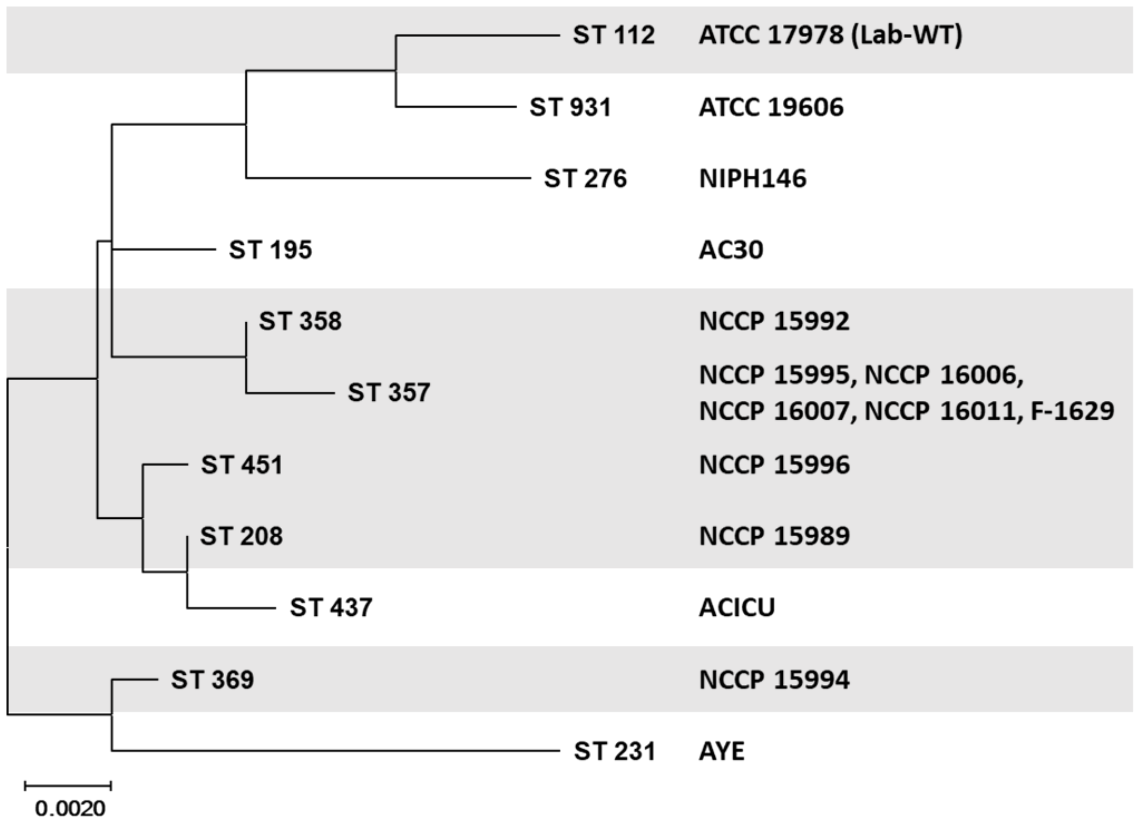

Supplementary Figure 1. Phylogenetic tree based on the seven housekeeping genes used in multilocus sequence typing analysis. The sequences of seven genes (*gltA*, *gyrB*, *gdhB*, *recA*, *cpn60*, *gpi*, and *rpoD*) used for multilocus sequence typing were combined to create a phylogenetic tree using the MEGA X program. For the phylogenetic tree, ATCC 19606, NIPH146, AC30, ACICU, and AYE were representative strains for ST931, ST276, ST195, ST437, and ST231, respectively. The gray background denotes strains used in this study.

Supplementary Figure 2.

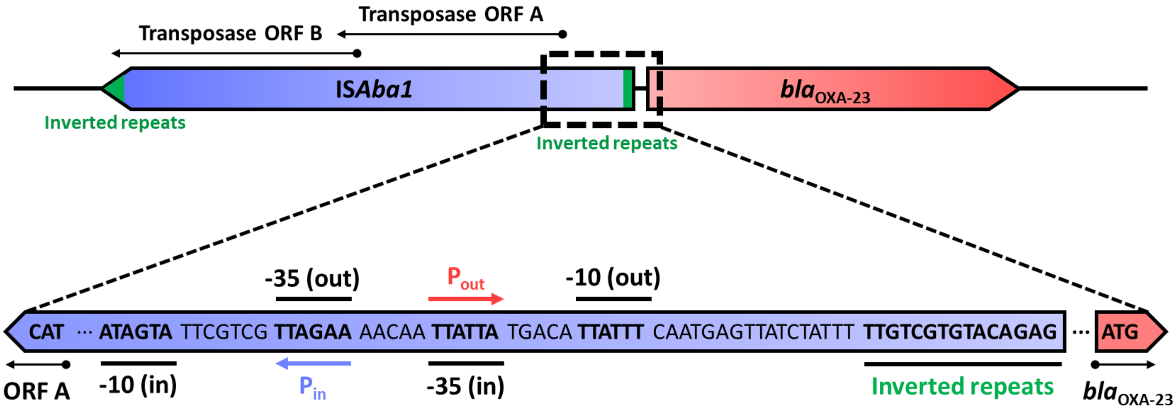

Supplementary Figure 2. Genetic schematic diagram of IS*Aba1*/*bla*OXA-23. The mobile genetic element IS*Aba1* contains two inverted repeat sequences and two transposase open-reading frames. The transposase inside IS*Aba1* is controlled by the P_{in} promoter, and the P_{out} promoter regulates the anti-sense gene in the region where IS*Aba1* is inserted. The *bla*$_{OXA-23}$ antibiotic resistance gene is regulated by the P_{out} promoter located upstream of IS*Aba1*, resulting in carbapenemase production.

Supplementary Figure 3.

a

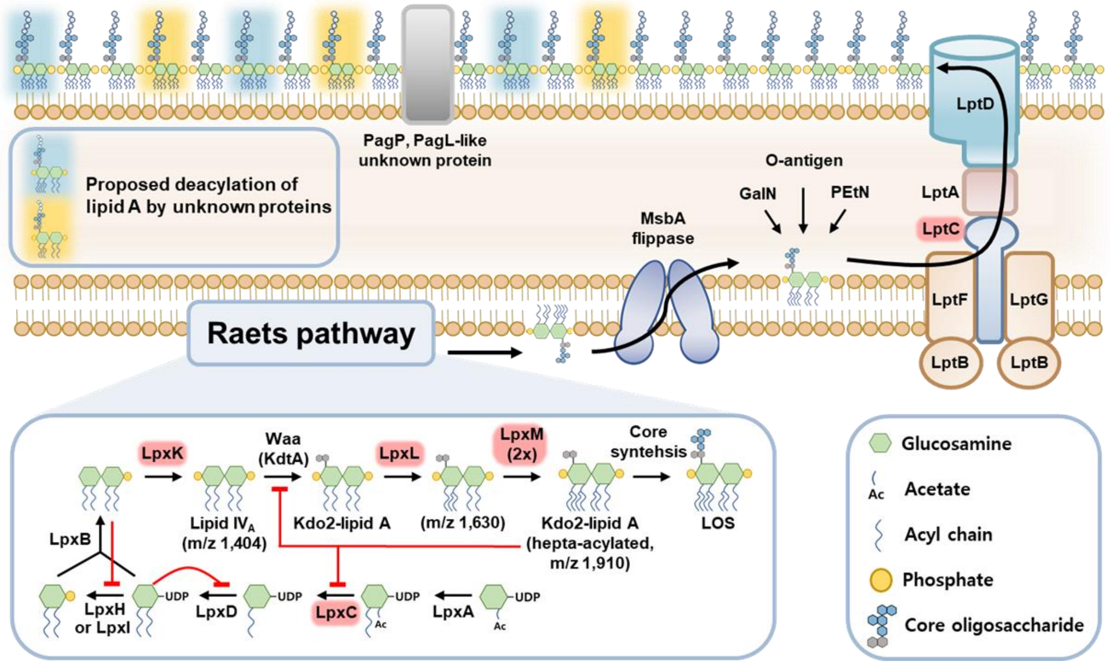

b

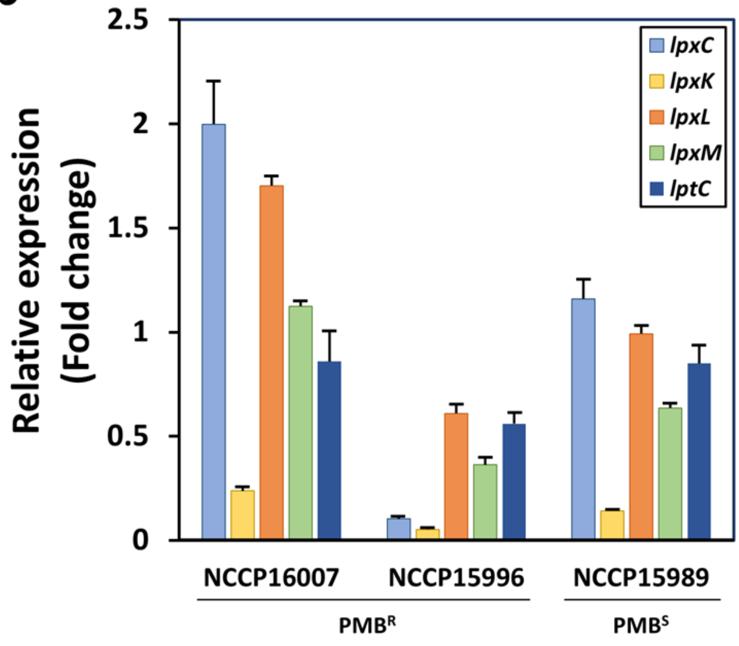

Supplementary Figure 3. Lipid A biosynthetic pathway. (a) Proposed biosynthesis and transport of *A. baumannii* lipooligosaccharide. Feedback inhibition is indicated by a red line. (b) Relative expression of LPS synthesis and transport pathway genes. qRT-PCR was performed in the same manner as that described in the Methods under the non-treated condition. The modification genes *lpxK*, *lpxL*, and *lpxM* are important genes involved in acylation during the formation of lipid A; however, their expression level according to PMB resistance has not been confirmed. PMB^R, PMB-resistant strains; PMB^S, PMB-susceptible strains.

Supplementary Figure 4.

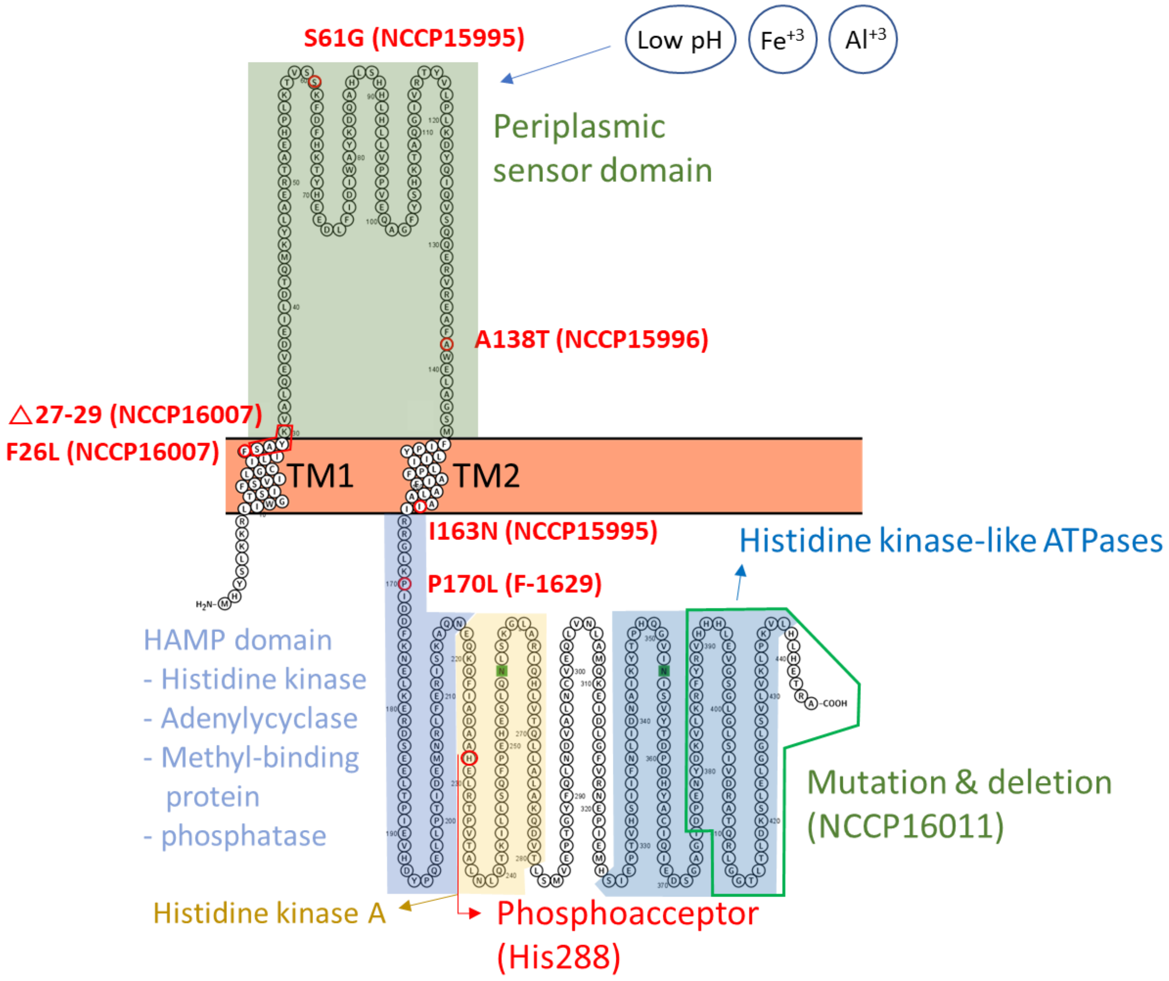

Supplementary Figure 4. PmrB mutation position on structure schematic. The domain of PmrB was analyzed using the web-based tool InterPro, and the transmembrane domain and peptide structure were analyzed using the web-based tool Potter. PmrB consists of two transmembrane domains and four functional domains: periplasmic sensor domain, HAMP domain, histidine kinase A, and histidine kinase-like ATPases. All mutations found in our PMB^R strains were located around the sensor domain. The deletion of amino acids at positions 27–29 in NCCP 16007 did not result in conformational changes in the transmembrane domain. In the case of the NCCP 16011 strain, it is presumed that the C-terminal part, which consists histidine kinase-like ATPases, was damaged by mutation and deletion and thus lost its function.

REFERENCES

1. Bartual, S. G., Seifert, H., Hippler, C., Luzon, M. A., Wisplinghoff, H., & Rodríguez-Valera, F. 2005. Development of a multilocus sequence typing scheme for characterization of clinical isolates of *Acinetobacter baumannii*. J Clin Microbiol 43:4382–4390.
2. Kim, M., Park, J., & Park, W. 2021. Genomic and phenotypic analyses of multidrug-resistant *Acinetobacter baumannii* NCCP 16007 isolated from a patient with a urinary tract infection. Virulence 12:150–164.
